# Optimization and Evaluation of Tourism Mascot Design Based on Analytic Hierarchy Process–Entropy Weight Method

**DOI:** 10.3390/e26070585

**Published:** 2024-07-09

**Authors:** Jing Wang, Fangmin Cheng, Chen Chen

**Affiliations:** School of Design, Xi’an Technological University, Xi’an 710021, China; chengfm1991@163.com (F.C.); 18391609257@163.com (C.C.)

**Keywords:** analytic hierarchy process–entropy weight, tourism mascot, design optimization, evaluation model

## Abstract

With the tourism industry continuing to boom, the importance of tourism mascots in promoting and publicizing tourism destinations is becoming increasingly prominent. Three core dimensions, market trend, appearance design, and audience feedback, are numerically investigated for deeply iterating tourism mascot design. Further, a subjective and objective evaluation weighting model based on the hierarchical analysis method (AHP) and entropy weighting method is proposed, aiming to utilize the advantages of these methods and ensure the entireness and correctness of results. Taking the mascots of six famous tourist attractions in Xi’an as an example, the feasibility and effectiveness of the evaluation model are verified. Data analysis and modeling results confirm that the three core evaluation indexes of scalability, innovation, and recommendation should be focused on in the design of tourism mascots in the three dimensions of market trends, appearance design, and audience feedback. The evaluation index scores are 0.1235, 0.1170, and 0.1123, respectively, which further illustrates the priority of mascot design. The evaluation model constructed by the research provides decision-makers with a comprehensive evaluation tool from the perspective of tourist experience, and also effectively assists the optimization process of mascot design. In addition, the model has good versatility and adaptability in structural design and evaluation logic and can be widely used in the optimization and evaluation research of brand mascots.

## 1. Introduction

The use of mascots for tourist attractions is of great significance for the development of cultural and creative brands and tourism brand marketing strategies. Through physical stores or online channels, this approach can effectively promote regional culture, special attractions, and major events [1]. For example, Japan, China, Thailand, Indonesia, and other places have adopted mascots for destination brand promotion; this suggests that mascots can be effective in increasing brand awareness, brand recognition, and consumer engagement, which can lead to beneficial consumer behavior [2]. However, the importance and promotional role of tourism mascots in the tourism industry have not yet received sufficient attention from the academic community [3]. The relevant mascot development literature suggests that product developers can benefit from marketing strategies and campaigns through tourism mascot development [3]. From a consumer perspective, this study argues that tourism mascots play a key role in regional economic development and brand image building. Therefore, we need to build a scientifically rigorous design evaluation system to deeply explore the development trend, appearance, and sensory evaluation of tourist mascots by consumers. Only when tourism mascots truly resonate with consumers and win their love and recognition can they fully realize their potential and drive the prosperity and development of regional culture and tourism economy.

The concept of mascot comes from the French word “Mascotte”, which refers to people, animals, or things that can bring good luck to people [1]. Mascots are usually endowed with rich symbolic meanings to meet people’s deep psychological needs. In terms of attracting public attention, cuteness is an important consideration in the design of mascots [4].

Tourism mascot design research involves many aspects, including mascot design principles, cultural connotations, marketing strategies, and their impact on tourism development. In terms of the value and design points of mascots, tourism mascots, as an important way to enhance the cultural content of tourist attractions, need to be designed in a way that reflects the relevance and expandability of the local culture [5]. The design should consider both image design and business promotion, combined with successful cases such as “Kumamoto Bears” to enhance the city’s popularity and economic benefits [6]. In addition, the design of the mascot should focus on the selection of prototypes and design methods to show a variety of shapes and personalized attributes. In terms of the role of mascots in tourism brand promotion, mascots have unique advantages in promoting the brand, attracting public attention, deepening people’s impression of the brand, and forming interactions between people and the brand. As for the key elements and ideas of mascot design, the key aspects of mascot design include demand positioning, emotional communication, and cultural identity [7]. To clarify the design thinking vein, design thinking should be organized based on these key elements. With the development of the information age, the aesthetic needs, design methods, dissemination channels, and scope of application of mascots have changed regarding the marketing and application of mascots. The creative marketing of mascots is important for the promotion of tourist destinations [8]. Meanwhile, the IP economy provides new insights into the design of rural tourism mascots, emphasizing the functions of three-dimensionality, interactivity, and diversification.

Radomskaya and Pierce [3] delve into the multiple roles of tourism mascots: “Tourism mascots are not only regional identity markers, cultural builders, and mediums of communication, but they are also tools for social engagement and carriers of information”. Based on this, the design of tourism mascots should be fully integrated with local cultural elements, and at the same time, it should be sufficiently attractive in terms of shape and color. A vivid, interesting, and attractive tourism mascot can not only present a friendly and approachable image but also effectively publicize the local culture and further promote economic development [9]. The above literature suggests that culture, brand, and regional symbols play a key role in tourism mascots. However, how to skillfully integrate these elements into the design to make it both representative and attractive is the key to establishing a reasonable tourism mascot design evaluation model.

Although many current studies have shown that tourism mascots play an important role in urban economic development cities, there are relatively few studies on the comprehensive evaluation of tourism mascot design. In addition, most research usually utilizes qualitative analysis or single-indicator analysis, which does not provide comprehensive evaluation results. In the process of designing and evaluating mascots, it is imperative to strike a balance between subjective and objective methodologies. Incorporating consumer emotional reactions and resonance while maintaining scientific rigor is imperative. To better capture the emotional reactions and resonance of consumers, designers must conduct thorough market research and consumer analysis. By comprehending consumer preferences, needs, and values, we can design mascots that more effectively meet their emotional demands. C.E. Shannon proposed the concept of information entropy in 1948 [10]. Based on the theory of information weighting, a weighting methodology that reflects the relative strength of the indices in crosstalk is proposed. The purpose of information entropy is fundamentally different from weighting methods. It is not about assigning weights to different elements of an electrical signal, but about quantifying the uncertainty or “messiness” of the information carried by those signals. In information theory, uncertainty is the likelihood of a random variable taking on different values, and information entropy is a measure of this uncertainty. Shannon’s information entropy formula is not a weighting method, but rather a tool for analyzing the probability distribution and uncertainty of electrical signals (or any other form of information). It provides us with a way to quantify the amount of information, allowing for us to gain a deeper understanding of the nature and character of information. It is widely used in multi-factor problems such as environmental evaluation [11], agricultural analysis [12], business judgment [13], etc. These studies show that the AHP weighting analysis method can scientifically evaluate this procedure.

The analytic hierarchy process (AHP) is a systematic analytic method that combines qualitative and quantitative analysis proposed by American operations research professor T.L. Saaty in the 1970 s [14,15]. It refers to a complex multi-objective decision-making problem as a system [16], with the decomposition of goals into multiple objectives or guidelines, which in turn are broken down into several levels of multiple indicators (or guidelines, constraints) [17]; it is a systematic method of calculating hierarchical single ranking (weights) and total ranking through fuzzy quantification of qualitative indicators to be used as a systematic method of decision-making for the optimization of objectives (multi-indicators) and multiple solutions [18]. This methodology has been applied in several fields, such as environmental and economic assessment [19], assessment of teaching quality [20], assessment of energy production technologies [21], etc. This method provides a basis for quantifying evaluation indexes and choosing the optimal scheme and has been extensively applied [22].

The AHP is a decision analysis method that combines qualitative and quantitative analysis with multi-objective and multi-criteria [23]. However, the relative importance of each factor is difficult to determine owing to the complexity of the specific implementation process. In response, researchers have proposed using fuzzy and rough numbers to deal with uncertainty in the scoring process, such as R-AHP, F-AHP, and other methods [24]. The entropy weight method reflects the utility value of the indicators and has a high correlation. AHP can handle qualitative indicators and system construction decision-making processes [25]. Therefore, the combination of the AHP and entropy weight method can be used for multivariate assessment problems with relatively independent indicators. The AHP–entropy weight method has been gradually accepted and recognized [26,27].

In this study, the AHP–entropy weight method was used for design evaluation to better analyze the design elements and functions of tourism mascots. The analytic hierarchy process can objectively analyze the design factors of mascots and requires a macro perspective to intervene and gradually subdivide to obtain solutions [28]. The current research on the evaluation of the design elements and characteristics of tourism mascots is not yet perfect; however, by using a combination of the hierarchical analysis method (AHP) and entropy weight method, we can assess the attractiveness of mascots more comprehensively and systematically. This approach can help us gain insight into the factors that are considered important in mascot design, thus providing strong support for the establishment of a complete set of evaluation index systems.

This study focuses on the tourism mascot as the core research object, combines the hierarchical analysis method and entropy weight method, and is committed to constructing a comprehensive and accurate evaluation model. The main contributions of this study can be summarized as follows: (1) In the current design practice, expert experience still occupies a prominent position in analyzing weights, whereas the application of systematic and complete extraction methods in design work is still insufficient. To address this issue, this paper is committed to constructing a comprehensive evaluation model based on hierarchical analysis and the entropy power method to optimize the design of tourism mascots, to achieve a more scientific and objective design decision. (2) This paper further delves into an optimization design strategy based on consumer preferences, aiming to refine mascot design elements that meet consumer expectations. This research not only provides an effective way for designers to create tourism mascots that are closer to market demand and satisfy consumers’ psychology but also provides a new perspective for the personalized and differentiated design of tourism products. (3) Enhancing the intrinsic cultural attributes of a product by evaluating key elements such as the exterior design of the tourism mascot. Simultaneously, these design elements can also enhance the perception, communication, and universality of cultural and creative products. Enhancing these design features is of great significance in promoting the prosperity of local tourism and enhancing the dissemination and influence of local culture. (4) The comprehensive evaluation model of tourism mascots developed in this study can fully consider the relative importance between different evaluation indexes as well as assign weights based on the information content of actual data, thus ensuring the completeness and accuracy of the evaluation results. The construction of this model not only provides a powerful tool to support the design, evaluation, and optimization of tourism mascots but also provides a valuable theoretical reference for the sustainable development and innovation of the tourism industry. Through the application of this method, we can more accurately grasp the needs of consumers and market trends and promote continuous innovation and development in the design field.

## 2. Research Methodology Framework

**Figure 1:**
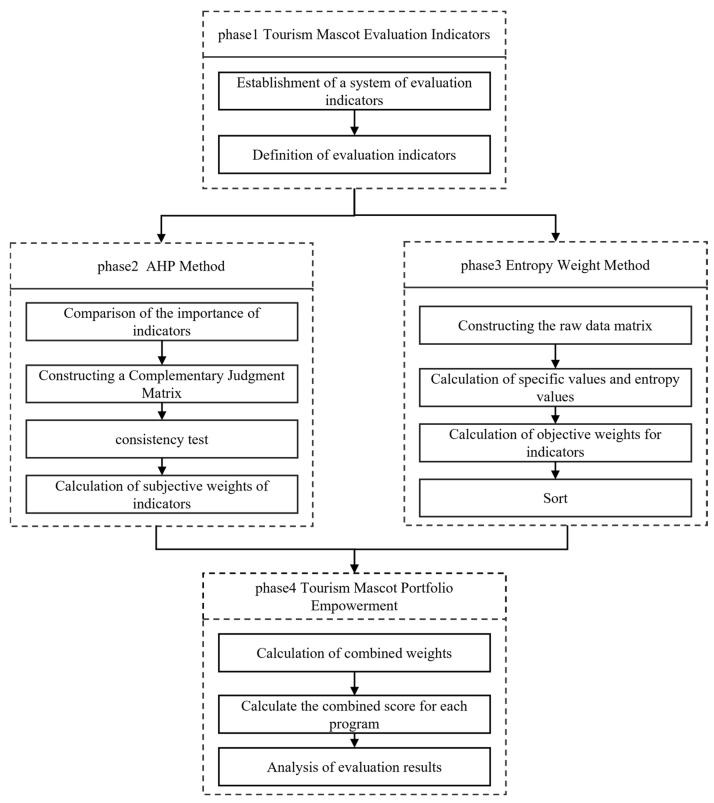
Research method framework based on AHP–entropy weight method.

Through the in-depth study of the hierarchical analysis method (AHP) and entropy weight method, we constructed a tourism mascot design evaluation model based on the AHP–entropy weight method [29]. The model can effectively reduce the subjectivity and uncertainty of the AHP method in dealing with evaluation indexes while simultaneously improving the accuracy of the quantitative analysis in the entropy weight method. The Tourism mascot evaluation model can scientifically evaluate design solutions, thus providing designers with a more reliable basis for decision-making. The steps of the tourism mascot evaluation model are as follows:

The first stage of the research and analysis of the key factors of tourism mascot design and development, through expert scoring assessment screening, can be used as a tourism mascot design assessment indicator. The second stage uses hierarchical analysis to compare the importance of indicators, construct a complementary judgment matrix, and calculate subjective evaluation weights. The third stage uses the entropy weighting method to construct the raw data matrix, and the objective evaluation weights of the indicators are derived by calculating the specific values and entropy values. The fourth part calculates the integrated evaluation weights and produces integrated evaluation results for each program. The specific process is shown in Figure 1 below.

### 2.1. Construction of an Evaluation Indicator System

#### 2.1.1. Establishment of a System of First-Rank Evaluation Indexes

During the development of a tourism mascot, designers must scrutinize its market performance and consumer response, as these factors are paramount in fostering emotional resonance with target audiences and ensuring market success. To accurately assess the design quality and market prospects of such mascots, an in-depth study was conducted with tourism industry experts spanning various geographical regions, resulting in the identification of three crucial indicators: market trends, appearance design, and audience feedback. These indicators not only offer designers a comprehensive understanding of market dynamics and consumer demands, but also provide specific guidelines for optimizing mascot design. By conducting rigorous data analysis and integrating expert opinions, designers can gain a more precise grasp of the design direction and market positioning of mascots, ultimately creating tourism mascots that are more captivating and competitive.

In terms of market trends, as the image representative of a tourist destination, the mascot’s cultural temperament and the experience of using the mascot are key factors in whether the mascot can survive continuously in the tourism market. Designers need to understand the market positioning, target customer groups of the tourism destination, and determine whether the mascot’s design style and image characteristics are compatible with the tourism destination’s positioning and brand image; at the same time, innovation, functionality, and comprehensibility should be fully considered to create a mascot image that meets market demand, thus guiding users to understand and purchase. Therefore, the tourist mascot market trend is considered the first-rank evaluation indicator A.

In terms of appearance design, the design of the mascot’s appearance needs to be consistent with the cultural connotation and brand characteristics of the tourist destination [30], especially the character modeling and color, etc., to convey the most intuitive feeling to the user. In the subsequent stages of dissemination, tourism mascots are also presented in the form of cultural symbols. Simplicity, easy recognition, and strong plasticity are the common characteristics of mascots that attract users’ attention. Therefore, uniqueness and recognizability are the only way to capture the audience’s attention and trigger their emotional resonance. During the development process, tourism agencies fully understand the brand image and audience needs, and skillfully integrate modern design with traditional elements to create a mascot image with distinctive characteristics and aesthetics. Therefore, the appearance design of tourism mascots is listed as the first-level evaluation indicator B.

In the dimension of audience feedback, audience feedback of a mascot is one of the important criteria for measuring its market performance and design quality. Excellent tourism mascots can shorten the emotional distance between consumers and establish a long-term emotional bond. Consumers gain emotional satisfaction through continuous purchasing behavior. Developers need to pay great attention to the audience’s evaluation of mascots and use market research, social media, and other channels to understand the audience’s feedback and evaluation on the mascot’s image, creativity, cultural connotation, etc.—by understanding the satisfaction, liking, and recommendation of the audience—and continuously optimize the design plan to improve the mascot’s recognition and market competitiveness. Satisfaction, liking, and recommendation can be independent in theory, but in practice they are usually interrelated. Satisfaction and liking usually influence recommendation, but recommendation can also be influenced by other factors. Therefore, the audience feedback of tourism mascots is used as the first-rank evaluation indicator C.

#### 2.1.2. Constructing the Evaluation Index System of Tourism Mascot

**Figure 2:**
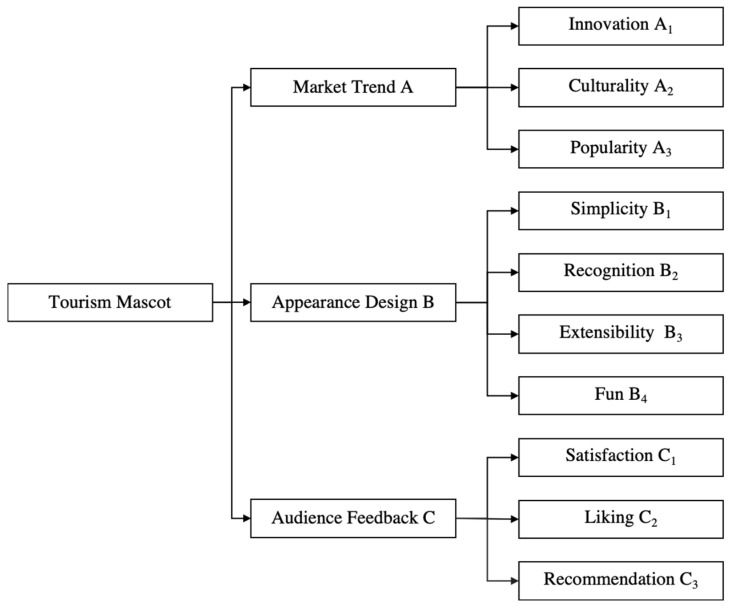
Tourism mascot evaluation index system.

By analyzing the elements of the evaluation indexes, three dimensions of market trend A, appearance design B, and audience feedback C were determined as the first-level indicators of the tourism mascots evaluation index. The development of the guideline layer defines criteria for each of the first-level evaluation indicators, from which representative evaluation factors are selected through the development of criteria. Based on the analysis of the composition of the first-level evaluation indicators of tourism mascot, innovation A_1_, culturality A_2,_ and popularity A_3_ were chosen as evaluation factors in the definition of the market trend dimension. Simplicity B_1_, recognition B_2_, extensibility B_3_, and fun B_4_ were chosen as evaluation factors in the definition of the appearance design dimension. Satisfaction C_1_, liking C_2_, and recommendation C_3_ were chosen as evaluation factors in the definition of the audience feedback dimension. A total of 10 evaluation factors are determined as the second-level indicators in the tourism mascot evaluation system. These 10 evaluation factors have been rigorously selected and objectively assessed by numerous tourism industry experts, ensuring the scientific rigor and effectiveness of the evaluation system. The tourism mascot evaluation index system is constructed, as shown in Figure 2.

Different evaluation indicators correspond to different cognitive characteristics. The explanations of the 10 selected secondary mascot evaluation indicators are shown in Table 1.

### 2.2. Determination of Evaluation Indicator Weights Based on the AHP Method

The AHP method is used to determine the weights of evaluation indicators, which mainly includes four steps: comparison of indicator importance, construction of complementary judgment matrix, consistency test, and calculation of weight values of each indicator.

Step 1: Construct a comparison matrix $\tilde{A}$ and invite *n* experts to assign language variables for pairwise comparison. The first-level indicators in the tourism mascot evaluation index system are compared pairwise on a scale of 1 to 9 to obtain the first-level indicator judgment matrix.

(1) $${\tilde{A}}_{D_{i}}^{k}=\left[\begin{array}{c} \begin{array}{c} 1\;\;\;\;\;\;{\tilde{a}}_{12}\;\;\;\;\cdots \;\;\;\;{\tilde{a}}_{1n}\\ {\tilde{a}}_{21}\;\;\;\;1\;\;\;\;\;\;\;\cdots \;\;\;\;\;{\tilde{a}}_{2n} \end{array}\\ \vdots \;\;\;\;\;\;\;\;\vdots \;\;\;\;\;\;\cdots \;\;\;\;\;\;\;\vdots \\ {\tilde{a}}_{n1}\;\;\;\;\;\;{\tilde{a}}_{n2}\;\;\;\cdots \;\;\;\;1\;\;\; \end{array}\right]$$

Here, we have the matrix $D_{i}\left(i=1,2\right)$, in which the *k*th evaluating user of ${\tilde{A}}_{D_{i}}^{k}$ compares the metrics to the dimensions.

To determine the relative importance of two comparative indicators, a pairwise comparison matrix is constructed for indicator A–C of the mascot.

Step 2: It is necessary to check the consistency of the matrix and the clarity of the indicator A–C pairwise comparison matrix. 

The fuzzy number $\tilde{M}\left(l,m,u\right)$ is converted to exact value by Equation (2). Then, ensure that the matrix fully satisfies consistency.

(2) $$M_{-crisp}=\left(l+4m+u\right)/6$$

Step 3: Consistency is checked to ensure that the matrices constructed from the consistency index CI and consistency ratio CR are consistent [31].

(3) $$\mathrm{CI}=\left(\lambda_{max}-n\right)/\left(n-1\right)$$

(4) CR=CI/RI

In Equation (3), λ_max_ is the maximum eigenvalue of matrix A and RI is the random indicator. When CR ≤ 0.1, the matrix is consistent; when CR > 0.1, the reverse is true [32].

Step 4: Determine the fuzzy weights of the indicators.

(5) $${\tilde{a}}_{ij}=\sqrt[{n}]{{\tilde{a}}_{ij}^{1}⊗{\tilde{a}}_{ij}^{2}⊗\cdots ⊗{\tilde{a}}_{ij}^{n}}$$

The geometric mean allows for determining the geometric mean and fuzzy weights of the indicators. Thus, it is possible to construct synthetic weights for the matrix of pairwise comparisons of *n* experts for indicators A–C.

For the determination of fuzzy geometric mean and fuzzy weights of indicators by geometric mean method, ${\tilde{r}}_{i}$ denotes the geometric mean of the fuzzy comparison values of indicator *i* with all the indicators *j*, ${\tilde{a}}_{ij}$ denotes the fuzzy comparison value from indicator *i* to *j* $\left(j=1,2,\cdots ,n\right)$, and ${\tilde{Y}}_{i}$ is the fuzzy weight of indicator *i*, which is denoted by the trigonometric fuzzy number ${\tilde{Y}}_{i}=\left(w_{i1},w_{i2},w_{in}\right)$.

(6) $${\tilde{r}}_{1}=\sqrt{\left({\tilde{a}}_{i1}⊗{\tilde{a}}_{i2}⊗\cdots ⊗{\tilde{a}}_{ij}\right)}$$

(7) $${\tilde{Y}}_{i}={\tilde{r}}_{i}⊗{\left({\tilde{r}}_{1}⊗{\tilde{r}}_{2}⊗\cdots ⊗{\tilde{r}}_{n}\right)}^{-1}$$

The fuzzy weights of each indicator in the first level evaluation of tourism mascots can be obtained through Equation (7).

### 2.3. Determination of Evaluation Indicator Weights Using the Entropy Weighting Method

This study extracts the design elements of tourism mascots based on consumer preferences. Therefore, it is more accurate to use the entropy weight method to determine the indicator weights [33,34]. The entropy weight method refers to the use of a decision matrix to find the entropy weight that represents the weight distribution. This method can efficiently avoid the influence of experts’ subjective judgment errors on the weights [35]. Entropy is an objective measure. For a certain indicator, its degree of dispersion can be judged by the entropy value. The smaller the value of information entropy, the greater the dispersion of the indicator, and the greater the influence of that indicator on the weight [36]. Therefore, the entropy weight method can provide a favorable basis for the comprehensive evaluation of multiple indicators [37]. This paper also uses this method to determine the weights of indicators.

The determination of evaluation index weights using the entropy weight method mainly includes the construction of the original data matrix, normalization processing, calculation of eigenvalues and entropy values, and calculation of evaluation index weights.

Invite *M* consumers to evaluate the tourism mascot on *m* sample programs, derive the evaluation data, select *n* evaluation indexes, and construct the original data matrix based on the evaluation $D={\left(d_{ij}\right)}_{m\times n}$:

(8) $$D={\left(d_{ij}\right)}_{m\times n}=\left[\begin{array}{c} d_{11}\;\;\;\;\;\;\;d_{12}\;\;\;\;\cdots \;\;\;\;d_{1n}\\ d_{21}\;\;\;\;\;\;\;d_{22}\;\;\;\;\cdots \;\;\;\;d_{2n}\\ \;\vdots \;\;\;\;\;\;\;\;\;\;\;\;\vdots \;\;\;\;\;\;\;\;\;\;\;\;\;\;\;\;\;\;\;\vdots \\ d_{m1}\;\;\;\;\;\;d_{m2}\;\;\;\;\;\cdots \;\;\;d_{mn} \end{array}\right]$$

To solve the problem of large differences in order of magnitude caused by the different evaluation indexes the matrix, D is normalized using Equations (9) and (10), and the standardized matrix $D^{\prime }={\left(d\prime_{ij}\right)}_{m\times n}$ is obtained. Equation (9) is a positive correlation formula, which applies to positive indicators, i.e., the larger the value of the evaluation indicator, the better; Equation (10) is a negative correlation formula, which applies to negative indicators, i.e., the smaller the value of the evaluation indicator, the better.

(9) $$d_{ij}^{\prime }=\frac{d_{ij}-\min d_{ij}}{\max d_{ij}-\min d_{ij}}$$

(10) $$d_{ij}^{\prime }=\frac{\max d_{ij}-d_{ij}}{\max d_{ij}-\min d_{ij}}$$

Calculating the characteristic weight of the evaluated value of the ith program under the jth evaluation indicator, that is, the value of the indicator weight of the jth evaluation indicator in the ith program, we obtain the matrix $F={\left(f_{ij}\right)}_{m\times n}$.

(11) $$f_{ij}=\frac{d_{ij}^{\prime }}{\sum_{j=1}^{n}d_{ij}^{\prime }}$$

Then, calculate the entropy value $E_{i}$ for the jth metric:

(12) $$E_{i}=-k\overset{n}{\underset{j=1}{\sum }}f_{ij}\mathrm{In}f_{ij},k=\frac{1}{\mathrm{In}n}$$

From the entropy value of the jth evaluation, the objective weight $W_{j}$ of the indicator can be calculated.

(13) $$W_{j}=\frac{1-E_{j}}{m-\sum_{j=1}^{m}E_{j}},\;0\leq W_{j}\leq 1,\;\overset{m}{\underset{j=1}{\sum }}W_{j}=1$$

### 2.4. Composite Weighting Calculation

The AHP is highly subjective when dealing with evaluation indicators, while the entropy weight method is mainly based on quantitative data and is more objective. Therefore, a combination of the AHP and entropy weighting method is used to assign weight values calculated separately. The comprehensive weight coefficient $\varphi_{j}$ of each evaluation indicator is obtained, which can make the experimental results more reasonable.

(14) $$\varphi_{j}=\frac{{\tilde{Y}}_{i}W_{j}}{\sum_{j=1}^{m}{\tilde{Y}}_{i}W_{j}},\;0\leq \varphi_{j}\leq 1,\;\overset{n}{\underset{j=1}{\sum }}\varphi_{j}=1$$

### 2.5. Calculation of Program Composite Score

The standardized eigenvalues of the *m* scenarios are weighted according to the composite weight coefficient $\varphi_{j}$ to obtain the final score *Q* of each scenario, and the scenarios are ranked accordingly.

(15) $$g_{ij}=\frac{d_{ij}}{\sum_{j=1}^{n}d_{ij}}$$

(16) $$Q_{i}=\varphi_{j}g_{ij}^{\prime }$$

## 3. Tourism Mascot Case Evaluation Application 

### 3.1. Determining the Subject of Evaluation

**Figure 3:**
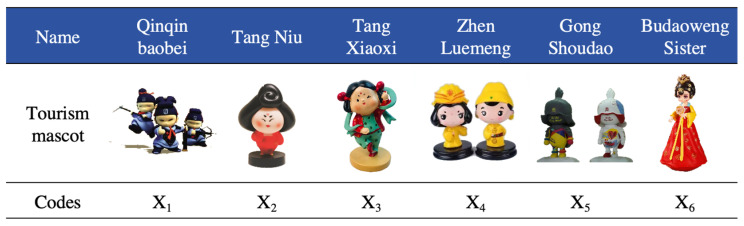
Tourism mascot cases.

This study is dedicated to evaluating the tourism mascots developed for various tourist attractions in Xi’an, Shaanxi Province, China. Based on an in-depth study of Xi’an’s regional culture, it conducts in-depth research on cultural and creative brands that integrate into Xi’an’s regional culture. Through actual visits to museums, tourist attractions, and cultural and creative stores; literature collection; field investigations; and interviews with local residents and tourists, a comprehensive review and analysis of the design of tourism mascots in the Xi’an area was conducted. Six research subjects were determined, namely: the Qinqin baobei, designed with the Terracotta Warriors and Horses of the Qin Shihuang Mausoleum Museum as the prototype; Tang Niu, designed with the Tang Dynasty lady figurines in the Shaanxi Provincial History Museum; Tang Xiaoxi, based on the figurines of ladies from the Xi’an Museum as the prototype; Tang Xiaodi and Tang Xiaofei, the representative of the Zhen Luemeng brand mascots, which were based on the poems of Li Longji, Emperor Xuanzong of the Tang Dynasty, in the “Book of Filial Piety”; the wall warriors designed with the Tang Dynasty Imperial Guards as the prototype in the Gong Shoudao series of cultural and creative products developed by the Xi’an City Wall; and the Sister Budaoweng, developed by the Datang City Scenic Area. These six groups of mascots are shown in Figure 3. The objects designed in this study were, respectively, coded as sample X_1_, X_2_, X_3_, X_4_, X_5_, and X_6_, and input into the tourism mascot evaluation model for identification and classification.

### 3.2. Determining the Evaluation Index Weights of Tourism Mascot Using the AHP Method

Two product design experts and three brand design experts were invited to form an expert panel to score the first-level and second-level indicators of tourism mascot design. On this basis, the expert group jointly examines the relative importance of the evaluation indicators in the experimental samples. We used a scale of 1–9 to make a qualitative comparison of the evaluation indicators and establish an indicator judgment matrix, as shown in Table 2. Then, we used SPSS (18.0) and Excel (2019) to solve the characteristic roots of the judgment matrix $M_{W}=\lambda_{\max_{w}}$, whose solution W^*^ (eigenvector) is the weight of each evaluation indicator at the same level relative to its previous indicator. According to Equations (3) and (4), a consistency test judgment matrix is constructed, then the weight of each evaluation indicator is calculated according to Equations (5)–(7), as shown in Table 2, Table 3, Table 4 and Table 5.

The weight of each indicator was calculated using the square root method. After normalization, the eigenvector (weight) of the judgment matrix A~C was W^*^ = (0.400, 0.233, 0.367). Further, the maximum eigenvalue of this vector was calculated to be λ_max_ = 3.000.

*CI* = (*λ_max_* − *n*)/(*n* − 1) = (3 − 3)/(3 − 1) = 0.000

*CR* = *CI/RI* = 0.000/0.520 = 0.000 < 0.1

It can be concluded that the results passed the consistency test and can be used for single-sorting work. Similarly, the judgment matrix and weights of each second-level indicator are calculated separately.

In summary, after integrating the indicators at each level the weight matrix of the final tourism mascot evaluation indicator system is constructed, as shown in Table 6 below.

### 3.3. Determination of Objective Weights for Second-Level Indicators Using the Entropy Weighting Method

Twenty-seven consumers were invited to score the six tourism mascots of Xi’an’s tourist attractions on their second-level indicators. The normalization was then performed according to Equations (9) and (10) to obtain the normalized matrix. Then, the entropy value of each evaluation indicator was calculated according to Equations (11) and (12), and the weight of the second-level index corresponding to each mascot was calculated from Equation (13). The results are shown in Table 7.

### 3.4. Comprehensive Weighting of Mascot Evaluation Indicators

The combined weight coefficients of the indicators are calculated according to Equation (14), and they are ranked as shown in Table 8.

After sorting, we obtained the importance of tourism mascot design evaluation indicators as follows: extensibility > innovation > recommendation > culturality > fun > simplicity > recognition > popularity > liking > satisfaction. Upon a thorough analysis of both subjective and objective evaluations, extensibility had the highest evaluation weight in the tourism mascot program, because the ductility of a mascot means that it is not a separate entity, but a basic symbol that can be reassembled and reconfigured. In comparison to other evaluation factors, this extensibility allows for tourism mascots to be reused in a variety of media and occasions, thereby increasing the exposure and influence of the product. Although the satisfaction of tourism mascots is important, it mainly depends on consumers’ emotional identification with and love for the mascot. Therefore, a good tourism mascot design can adapt to different cultures, times, and contexts, ensuring the effective transmission of cultural information, and extensibility can effectively help tourism mascots to better perform this function.

### 3.5. Calculating the Overall Score of the Tourism Mascot

According to Equations (15) and (16), the standardized eigenvalues of the six scenarios are weighted according to the integrated weight coefficient φ and the comprehensive score of each scenario can be obtained, as shown in Table 9.

As can be seen from Table 9, the ranking of the comprehensive scores of each program is X_6_ > X_5_ > X_2_ > X_1_ > X_3_ > X_4_, so X_6_ can be determined as the best scheme, and X_6_ will be used as a reference prototype in subsequent related designs. After analyzing the opinions of the design experts, it is believed that the X_6_ scheme has a beautiful and lovely shape, and the expression of combining Tang Dynasty cultural elements with the tumbler is relatively novel in the current tourism mascot design. The mascot has rich body language, vivid expression, smooth lines, and coordinated colors. In addition, the costumes and props with a strong Tang Dynasty cultural atmosphere make the character modeling very exquisite. Combining the subjective analysis of experts with the objective statistics of data can provide a more systematic and comprehensive reference for the research, and at the same time verify the reliability of the evaluation results.

By analyzing the above experiments and related literature, and combining the feedback from design experts and consumers, it can be seen that the market trend has a greater impact on the innovation factors in mascot design, the appearance design has a greater impact on the extension design of the mascot, and the audience feedback is most affected by the recommendation degree of the tourism mascot.

## 4. Discussion

According to the comprehensive evaluation score in Table 9, the evaluation results of X_6_ > X_5_ > X_2_ > X_1_ > X_3_ > X_4_ can be obtained, and combined with the objective evaluation in Table 7, the evaluation results of each program design are analyzed as follows:

X_1_ uses the Terracotta Warriors as design elements. The Terracotta Warriors are highly recognizable and have a distinctive style. They can transcend different cultures and age groups and have international popularity. Therefore, they are rated the highest in terms of scalability. The reason for the low satisfaction score is mainly because the audience has high expectations for the mascot, and the actual effect of the mascot just meets the audience’s lowest expectations, resulting in a low satisfaction score.

X_2_ scores the highest in terms of ductility, mainly because it adopted the Tang Dynasty ladies’ modeling design, which contains historical and cultural elements, is highly recognizable, can be presented in a variety of forms, and has strong scalability. However, the Tang Nui’s design rarely considers modern aesthetic trends, resulting in a gap between simplicity and modern aesthetic standards.

X_3_ uses Tang Dynasty cultural elements as design elements, and its image is cute, friendly, and fun, which brings positive values to consumers and makes them more willing to share and spread it. Therefore, X_3_ scores the highest in terms of recommendation. However, compared with Tang Niu, Tang Xiaoxi’s image lacks effective brand publicity and promotion, and its visibility is limited, resulting in low recognition.

The design of X_4_ is inspired by the images of Emperor Tang Xuanzong of Li Longji and Yang Yuhuan. The color scheme is mainly dignified yellow. The overall shape is clean and neat, with the highest score in simplicity. However, the shape is not novel enough and is too ordinary. The design elements are not prominent enough, and the satisfaction is low.

X_5_ is designed with visual elements of the imperial road of the Tang Dynasty. It uses a simple, strong, and resolute visual expression to highlight the message characteristics of a battle-hardened, mighty, and majestic soldier. It has the highest innovation score but is not suitable for presentation in other scenes and media. It also has low recognition and identification in other cultural activities, and a low extensibility score.

X_6_ has more design elements than other mascots in terms of form, expression, color, and decoration. It also has the characteristics of a tumbler and is rated highest for its innovation. However, excessive exposure can lead to aesthetic fatigue and lack of freshness, so people gradually lose interest and popularity decreases.

Furthermore, the AHP–entropy weight evaluation index model constructed in this study uses market trends, appearance design, and audience feedback as the first-level evaluation index system to evaluate the mascots of six different scenic spots and obtain the optimal solution through comprehensive scoring. The following is an analysis from the first-level index dimension:

In terms of the market trend dimension, the top two X_6_ and X_5_ in the statistical composite score, both used Tang cultural elements in their designs. Tang culture has become one of the important regional cultural representatives of Xi’an’s regional culture. On the one hand, Tang was a dynasty with highly developed economy, culture, science and technology, and art. It promoted exchanges between ethnic groups, absorbed foreign cultures, and formed a rich and diverse cultural form. Tang culture plays a key role in aesthetics. On the other hand, Tang culture conforms to the inclusive and open character of today’s social culture. Including the current popularity of Tang clothing, accessories, etc., it is easy for it to be loved and recognized by consumers. Qin culture should take the Terracotta Warriors and Horses of Qin as the prototype, deeply explore the core elements of cultural vision, and combine with future market development trends to create a better tourism mascot.

From the perspective of appearance design, modeling elements are the key elements of tourism mascot design, which can give consumers the most profound and direct sensory experience. Ingeniously integrating regional cultural elements into the appearance design of mascots can not only allow for consumers to unconsciously accept the connotations of regional culture through the dual experience of visual and touch, but also significantly enhance the creative effect and attractiveness of tourism mascots. In order to better show the characteristics of regional culture and convey its profound connotation, the creation of mascots should be closely linked to the characteristics of the expression to be conveyed, and carefully designed in combination with the beauty of form. For example, the mascot X_6_’s form has some lifelike dynamics added, which is lively and gives people a comfortable and friendly feeling. Therefore, an excellent mascot form is a combination of the object’s “shape” and “demeanor”, achieving “both form and spirit”.

From the perspective of audience feedback, excellent tourism mascot design should not be limited to simple improvements on historical figures and natural environments. Instead, it should explore deeper interactions with humanistic and memory, and stimulate consumers’ emotional resonance and aesthetic experience. Such mascot design can touch consumers’ hearts and trigger their nostalgia for good memories, and thus generates affinity and happiness. For example, the mascot X_6_ has a casual and relaxed demeanor, a free and lively image, and is full of life interest. It is easy to bring young consumers closer and generate empathy. This emotional connection not only strengthens the bond between the mascot and consumers, but also gives the tourism mascot deeper cultural value and social significance. Therefore, incorporating emotional elements into mascot design is the key to achieving cultural communication and emotional resonance.

## 5. Conclusions

The design elements analyzed and extracted from the regional culture of Xi’an can be used to both realize its cultural heritage and meet the needs of the market. To highlight the regional cultural characteristics of Xi’an, each scenic spot in Xi’an has incorporated its tourism mascot with its distinctive cultural elements. To study the influence of market trends, appearance design, and audience feedback on the design of tourism mascots, this research adopted a combination of the AHP and entropy power method. The representative tourist mascot designs of six famous tourist attractions in Xi’an were evaluated to reduce the influence of subjective factors in the design evaluation process and to ensure the credibility and accuracy of the evaluation results. An evaluation index system for tourism mascots was constructed, and based on the hierarchical analysis method and entropy weighting method, combined with combination assignment and weighting calculation, the comprehensive weight calculation method of the evaluation index based on consumer scoring was summarized. The comprehensive scores of six scenic tourist mascots were obtained using the evaluation indexes’ comprehensive weights, and the optimal program was screened out to improve the objectivity and accuracy of the evaluations.

1. Objectivity and science: In the design process of tourism mascots, priority should be given to the three evaluation indicators of extensibility, innovation, and recommendation. The weights of these three evaluation indicators were the highest, which were 0.1235, 0.1170, and 0.1123, respectively. The design of tourism mascots can be ranked according to the weight the evaluation index system. This method reduces the influence of subjective judgment, making the evaluation results more objective and scientific. Targeted strategies to improve the design level of tourism mascots can effectively enhance the influence of tourism mascots.
2. Comprehensive and multidimensional evaluation: The AHP method decomposes complex decision-making problems into multiple levels and factors by constructing a hierarchical model, which is easy to understand and operate. Combined with the entropy method, it can effectively handle the uncertainty and randomness in the data and improve the evaluation accuracy. This comprehensive evaluation method can comprehensively consider all aspects of tourism mascot design, such as creativity, cultural connotation, market potential, etc., and make more scientific and reasonable evaluation and optimization suggestions.
3. Flexibility and applicability: The application scope of the AHP–entropy weight method is very wide. It can be used not only for economic benefit evaluation, but also for tourism value assessment, sustainable development evaluation, tourism planning evaluation, and many other fields. This study applied the results of the AHP–entropy weight method to the optimization of tourism mascot design schemes. The proposed tourism mascot design evaluation model can help designers make reasonable decisions and provide a reference for design practice and other mascot designs. At the same time, the method is flexible and widely applicable, which can meet the needs of different fields and problems.

This research on tourism mascot design optimization evaluation using the AHP–entropy weight method also has certain limitations, such as poor data quality or insufficient data volume having affected the accuracy and reliability of the evaluation; the complexity and computational volume of the evaluation process; and no clear solution for how to properly integrate the information between expert experience and objective data in practice.

In summary, the tourism mascot design optimization and evaluation research based on the AHP–entropy weight method has the advantages of strong comprehensiveness, objectivity, and high scientificity. However, it also faces limitations such as high data quality requirements, high computational complexity, and difficulty in balancing expert experience and objective data. Therefore, in practical application, it is necessary to flexibly select and adjust the evaluation method according to specific situations to ensure the effectiveness and practicality of the evaluation.

## Figures and Tables

**Table 1:** Evaluation index and descriptions.

Indicator	Indicator Description and Reference
Innovation A_1_	Fully explore the enterprise or regional cultural connotation and commercial value and based on this unique and creative design.
Culturality A_2_	Reflecting specific cultural values and traditions in the design process, cultural elements are integrated into the design of the mascot so that it has a deep cultural heritage and distinctive cultural characteristics.
Popularity A_3_	The image and characteristics of the mascot are combined with the current popular culture, aesthetic trends, and social background so that they are accepted and loved by the public.
Simplicity B_1_	Express the qualities of the mascot design and the brand message it represents most simply and intuitively.
Recognition B_2_	Highly personalized and unique, easily recognized and remembered by consumers.
Extensibility B_3_	It can be recombined as a basic element and become an easy-to-circulate communication medium that is easily accepted by and emotionally resonates with consumers.
Fun B_4_	Approachable, attractive, and able to attract the attention and interest of consumers.
Satisfaction C_1_	Able to meet the aesthetic needs of consumers.
Liking C_2_	It can be loved and recognized by consumers.
Recommendation C_3_	Being able to receive recommendations from consumers.

**Table 2:** Indicator judgment matrix.

	Market Trend A	Appearance Design B	Audience Feedback C	W^*^
Market trend A	1	1.714	1.091	0.4000
Appearance design B	0.583	1	0.636	0.2333
Audience feedback C	0.917	1.571	1	0.3667
λ_max_ = 3; CI = 0.000; RI = 0.520; CR = 0.000 < 0.10				

**Table 3:** Evaluation index A.

	A_1_	A_2_	A_3_	W^*^
A_1_	1	1.375	1	0.3667
A_2_	0.727	1	0.727	0.2666
A_3_	1	1.375	1	0.3667
λ_max_ = 3; CI = 0.000; RI = 0.520; CR = 0.000 < 0.10				

Conclusion: The CR value was less than 0.10 and passed the consistency test.

**Table 4:** Evaluation index B.

	B_1_	B_2_	B_3_	B_4_	W^*^
B_1_	1	1.100	0.579	1.100	0.2200
B_2_	0.909	1	0.526	1	0.2000
B_3_	1.727	1.900	1	1.900	0.3800
B_4_	0.909	1	0.526	1	0.2000
λ_max_ = 4; CI = 0.000; RI = 0.890; CR = 0.000 < 0.10					

Conclusion: The CR value was less than 0.10 and passed the consistency test.

**Table 5:** Evaluation index C.

	C_1_	C_2_	C_3_	W^*^
C_1_	1	0.667	0.400	0.2000
C_2_	1.500	1	0.600	0.3000
C_3_	2.500	1.667	1	0.5000
λ_max_ = 3; CI = 0.000; RI = 0.520; CR = 0.000 < 0.10				

Conclusion: The CR value was less than 0.10 and passed the consistency test.

**Table 6:** Tourism mascot evaluation index system subjective weights.

Estimation	W^*^	First-Level Indicators	W^*^	Second-Level Indicators	W^*^	Final Subjective Weights
Tourism mascot evaluation	1	Market trend A	0.4000	Innovation A_1_	0.3667	0.1468
				Culturality A_2_	0.2666	0.1064
				Popularity A_3_	0.3667	0.1468
		Appearance design B	0.2333	Simplicity B_1_	0.2200	0.0513
				Recognition B_2_	0.2000	0.0466
				Extensibility B_3_	0.3800	0.0885
				Fun B_4_	0.2000	0.0466
		Audience feedback C	0.3667	Satisfaction C_1_	0.2000	0.0734
				Liking C_2_	0.3000	0.1101
				Recommendation C_3_	0.5000	0.1835

**Table 7:** Weights of evaluation factors for second-level indicators of tourism mascots.

Serial Number	Second-Level Indicators	X_1_	X_2_	X_3_	X_4_	X_5_	X_6_
A_1_	Innovation	0.1172	0.0641	0.0981	0.0762	0.1556	0.1909
A_2_	Culturality	0.0672	0.0812	0.1251	0.0987	0.1214	0.1161
A_3_	Popularity	0.0717	0.1325	0.0878	0.1205	0.0900	0.0694
B_1_	Simplicity	0.1231	0.0587	0.0727	0.1259	0.0878	0.1243
B_2_	Recognition	0.0855	0.1400	0.0579	0.0994	0.1142	0.0899
B_3_	Extensibility	0.1673	0.1667	0.1056	0.1242	0.0545	0.1225
B_4_	Fun	0.1317	0.0743	0.1081	0.0885	0.1428	0.0602
C_1_	Satisfaction	0.0456	0.0615	0.0766	0.0757	0.0954	0.0970
C_2_	Liking	0.1309	0.0632	0.0732	0.0921	0.0611	0.0445
C_3_	Recommendation	0.0598	0.1578	0.1949	0.0988	0.0772	0.0852

**Table 8:** Comprehensive weight of evaluation indexes for tourism mascot.

Serial Number	Second-Level Indicators	W^*^	Sort
A_1_	Innovation	0.1170	2
A_2_	Culturality	0.1016	4
A_3_	Popularity	0.0953	8
B_1_	Simplicity	0.0988	6
B_2_	Recognition	0.0978	7
B_3_	Extensibility	0.1235	1
B_4_	Fun	0.1009	5
C_1_	Satisfaction	0.0753	10
C_2_	Liking	0.0775	9
C_3_	Recommendation	0.1123	3

**Table 9:** Comprehensive score of each scheme.

Tourism Mascot	X_1_	X_2_	X_3_	X_4_	X_5_	X_6_
Comprehensive Score	0.1007	0.1012	0.1005	0.1003	0.1014	0.1016
Sort	4	3	5	6	2	1

## Data Availability

The data used to support the findings of this study are included in the article.

## References

[1] Schattschneider E. (2005). The Bloodstained Doll: Violence and the Gift in Wartime Japan. J. Jpn. Stud..

[2] Patterson A., Khogeer Y., Hodgson J. (2013). How to create an influential anthropomorphic mascot: Literary musings on marketing, make-believe, and meerkats. J. Mark. Manag..

[3] Radomskaya V., Pearce P.L. (2021). Adding character: The role of destination mascots in tourism development. Tour. Manag..

[4] Xu J., Yan L., Pratt S. (2022). Destination image recovery with tourism mascots. J. Destin. Mark. Manag..

[5] Lei S. (2019). The value and design of cultural and tourism mascots. Art Obs..

[6] Zhixin L. (2021). Research on the Design of Tourism Mascot in Ji’an City—Combining the Successful Experience of Kumamoto Kumamoto in Japan. Cult. J..

[7] Li N., Jin G.-F. (2016). Study on The Essential Design Factors and Ideas of Mascot. Art Des..

[8] Chen L. (2020). The New Trend of Mascot Design in the Information Age. Packag. Eng..

[9] Su Q., Li F. (2023). How cute mascots affect relationships with tourism destinations: A moderated mediation model. Tour. Manag..

[10] Ma Z., Qin S., Cao C., Lv J., Li G., Qiao S., Hu X. (2019). The Influence of Different Knowledge-Driven Methods on Landslide Susceptibility Mapping: A Case Study in the Changbai Mountain Area, Northeast China. Entropy.

[11] Zhou R., Pan Z., Jin J., Li C., Ning S. (2017). Forewarning Model of Regional Water Resources Carrying Capacity Based on Combination Weights and Entropy Principles. Entropy.

[12] Hou M., Lin Z., Chen J., Zhai Y., Jin Q., Zhong F. (2018). Optimization on theBuried Depth of Subsurface Drainage under Greenhouse Condition Based on Entropy Evaluation Method. Entropy.

[13] Li M., Wang J., Li Y., Xu Y. (2018). Evaluation of Sustainability Information Disclosure Based on Entropy. Entropy.

[14] Saaty T.L. (1977). A scaling method for priorities in hierarchical structures. J. Math. Psychol..

[15] Saaty T.L. (1980). The analytic hierarchy process (AHP). J. Oper. Res. Soc..

[16] Xu X., Zhang Z., Yuan J., Shao J. (2023). Design and multi-objective comprehensive evaluation analysis of PV-WT-BG-Battery hybrid renewable energy systems in urban communities. Energy Convers. Manag. X.

[17] Feng G., Lei S., Guo Y., Meng B., Jiang Q. (2019). Optimization and Evaluation of Ventilation Mode in Marine Data Center Based on AHP-Entropy Weight. Entropy.

[18] Luo C., Lu Z., Gong Y., Ma Z. (2017). The Comprehensive Evaluation of Optimization Air-Condition System Based on Analytic Hierarchy Methodology. Energy Procedia.

[19] Zhang L., Lavagnolo M.C., Bai H., Pivato A., Raga R., Yue D. (2019). Environmental and economic assessment of leachate concentrate treatment technologies using analytic hierarchy process. Resour. Conserv. Recycl..

[20] Shen L., Yang J., Jin X., Hou L., Shang S., Zhang Y. (2019). Based on Delphi method and Analytic Hierarchy Process to construct the Evaluation Index system of nursing simulation teaching quality. Nurse Educ. Today.

[21] Kheybari S., Rezaie F.M., Naji S.A., Najafi F. (2019). Evaluation of energy production technologies from biomass using analytical hierarchy process: The case of Iran. J. Clean. Prod..

[22] Wu Y. (2023). Evolutionary design method for transforming ceramic cultural elements into cultural and creative products. J. Intell. Fuzzy Syst..

[23] Yu C.M.M. (2022). Evaluation of Paralleling Machine Design Based on AHP and Weighted Grey Association Analysis. Packag. Eng..

[24] Sun C.-C. (2010). A performance evaluation model by integrating fuzzy AHP and fuzzy TOPSIS methods. Expert Syst. Appl..

[25] Dos Santos P.H., Neves S.M., Sant’Anna D.O., Oliveira C.H.d., Carvalho H.D. (2019). The analytic hierarchy process supporting decision making for sustainable development: An overview of applications. J. Clean. Prod..

[26] Li L., Liu F., Li C. (2014). Customer satisfaction evaluation method for customized product development using Entropy weight and Analytic Hierarchy Process. Comput. Ind. Eng..

[27] Chen T., Jin Y., Qiu X., Chen X. (2014). A hybrid fuzzy evaluation method for safety assessment of food-waste feed based on entropy and the analytic hierarchy process methods. Expert Syst. Appl..

[28] Luo S.-J., Zhu S.-S., Feng C. (2008). Product Family Design DNA in Industrial Design. Chin. J. Mech. Eng..

[29] Yang Y., Shi Y., Wang T. A Blockchain technology application maturity assessment model for digital government public service projects. Proceedings of the 5th International Conference on Crowd Science and Engineering.

[30] Tang B., Jiang L., Zhang X., Fu R., Yang S. Mobile Platform Type Lower Limb Rehabilitation Electromechanical System. Proceedings of the 2020 International Wireless Communications and Mobile Computing (IWCMC).

[31] Chung S.-H., Lee A.H.I., Pearn W.L. (2005). Product Mix Optimization for Semiconductor Manufacturing Based on AHP and ANP Analysis. Int. J. Adv. Manuf. Technol..

[32] Chen X., Wenjia Z. (2023). Support carbon neutrality target—Which flexible power source is the best option for China?. Energy.

[33] Amiri V., Rezaei M., Sohrabi N. (2014). Groundwater quality assessment using entropy weighted water quality index (EWQI) in Lenjanat, Iran. Environ. Earth Sci..

[34] Li L., Liu D.-J. (2014). Study on an Air Quality Evaluation Model for Beijing City Under Haze-Fog Pollution Based on New Ambient Air Quality Standards. Int. J. Environ. Res. Public Health.

[35] Hu Y., Yu S., Qin S., Chen D., Chu J., Yang Y. (2021). How to extract traditional cultural design elements from a set of images of cultural relics based on F-AHP and entropy. Multimed. Tools Appl..

[36] Wang M., Ye C., Zhang D. (2022). Evaluation of Green Manufacturing Level in China&rsquo;s Provincial Administrative Regions Based on Combination Weighting Method and TOPSIS. Sustainability.

[37] Gong F., Xiang S., Chen J., Wang Y. Big data evaluation model of football team cooperation based on entropy weight method. Proceedings of the 2020 7th International Conference on Information, Cybernetics, and Computational Social Systems (ICCSS).

